# Nanomedicine based on chemotherapy-induced immunogenic death combined with immunotherapy to enhance antitumor immunity

**DOI:** 10.3389/fphar.2024.1511423

**Published:** 2024-12-04

**Authors:** Yichang Chen, Kuirong Mao, Dongxiao Han, Ruolin Ma, Tianmeng Sun, Haipeng Zhang, Bing Han

**Affiliations:** ^1^ Department of Breast Surgery, General Surgery Center of The First Hospital, Jilin University, Changchun, China; ^2^ Key Laboratory of Organ Regeneration and Transplantation of Ministry of Education, The First Hospital, Institute of Immunology, Jilin University, Changchun, China; ^3^ National-local Joint Engineering Laboratory of Animal Models for Human Diseases, Changchun, China; ^4^ Department of Breast Surgery, Zhengzhou Central Hospital Affiliated to Zhengzhou University, Zhengzhou, Henan, China; ^5^ International Center of Future Science, Jilin University, Changchun, China; ^6^ Department of Gynecology, Obstetrics and Gynecology Center, The First Hospital of Jilin University, Changchun, China

**Keywords:** breast cancer, tumor immunogenic cell death, combination immunotherapy, nanomedicine, antitumor immunity

## Abstract

**Introduction:**

Chemo-immunotherapy based on inducing tumor immunogenic cell death (ICD)with chemotherapy drugs has filled the gaps between traditional chemotherapy and immunotherapy. It is verified that paclitaxel (PTX) can induce breast tumor ICD. From this basis, a kind of nanoparticle that can efficiently deliver different drug components simultaneously is constructed. The purpose of this study is for the sake of exploring the scheme of chemotherapy-induced ICD combined with other immunotherapy to enhance tumor immunogenicity and inhibit the growth, metastasis, and recurrence of breast tumors, so as to provide a research basis for solving the tough problem of breast cancer treatment.

**Methods:**

Nanomedicine loaded with PTX, small interference RNA that suppresses CD47 expression (CD47siRNA, siCD47), and immunomodulator R848 were prepared by the double emulsification method. The hydrodynamic diameter and zeta potential of NP/PTX/siCD47/R848 were characterized. Established the tumor-bearing mice model of mouse breast cancer cell line (4T1) *in situ* and observed the effect of intravenous injection of NP/PTX/siCD47/R848 on the growth of 4T1 tumor *in situ*. Flow cytometry was used to detect the effect of drugs on tumor immune cells.

**Results:**

NP/PTX/siCD47/R848 nano-drug with tumor therapeutic potential were successfully prepared by double emulsification method, with particle size of 121.5 ± 4.5 nm and surface potential of 36.1 ± 2.5 mV. The calreticulin on the surface of cell membrane and extracellular ATP or HMGB1 of 4T1 cells increased through treatment with NPs. NP/PTX-treated tumor cells could cause activation of BMDCs and BMDMs. After intravenous injection, NP/PTX could quickly reach the tumor site and accumulate for 24 h. The weight and volume of tumor *in situ* in the breast cancer model mice injected with nanomedicine through the tail vein were significantly lower than those in the PBS group. The ratio of CD8^+^/CD4^+^ T cells in the tumor microenvironment and the percentage of dendritic cells in peripheral blood increased significantly in breast cancer model mice injected with nano-drugs through the tail vein.

**Discussion:**

Briefly, the chemotherapeutic drug paclitaxel can induce breast cancer to induce ICD. The nanomedicine which can deliver PTX, CD47siRNA, and R848 at the same time was prepared by double emulsification. NP/PTX/siCD47/R848 nano-drug can be enriched in the tumor site. The experiment of 4T1 cell tumor-bearing mice shows that the nano-drug can enhance tumor immunogenicity and inhibit breast tumor growth, which provides a new scheme for breast cancer treatment. (Graphical abstract)

## Graphical Abstract

**Figure:**
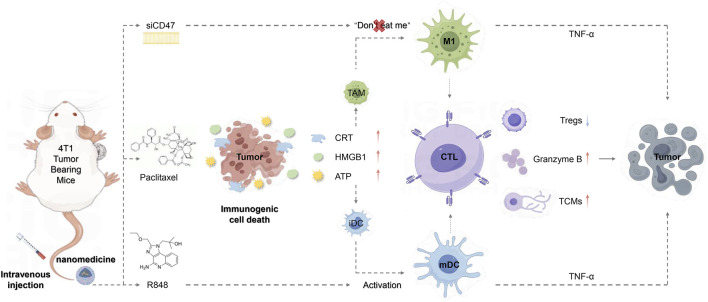
Intravenous injection of PTX-loaded nanomedicine based on PEG-PLGA causes immunogenic death of tumor cells and simultaneously reduces tumor cell immune escape, promotes macrophage polarization towards MI subtypes, and effectively activates DCs, thereby enhancing anti-tumor immunity.

## 1 Introduction

As the second leading cause of cancer death in women, breast cancer remains the most common one, while the primary reasons of them are distant metastasis and recurrence (Giaquinto et al., 2022). Chemotherapy and surgery are still the main treatments for breast cancer up to now, yet the effectiveness of chemotherapy is hindered by multidrug resistance, recurrence, and metastasis (Duan et al., 2023; Zhang et al., 2021). In recent years, immunotherapy has become a new strategy for various tumor treatments (Savas et al., 2016). However, breast cancer itself is a cold tumor with low immunogenicity, single drug immunotherapy cannot obtain an effective anti-tumor immune response (Si et al., 2021). Tumor immunogenic cell death (ICD) can not only transform cancer into a “therapeutic vaccine” to make dying cancer cells immunogenic, but also trigger an immune response by activating dendritic cells (DC) and then activating specific T cell response (Wu et al., 2021). Some traditional chemotherapeutic drugs can induce ICD. This chemical immunotherapy makes up for the shortcomings of conventional chemotherapy and immunotherapy (Wang et al., 2022).

High expression of CD47 in breast cancer enables tumor cells to escape the attack of the innate immune system, which is closely related to the low immunogenicity of tumors (Jia et al., 2021). Inhibition of CD47 expression can enhance the anti-tumor immunity of breast cancer (Folkes et al., 2018; Lentz et al., 2021). Toll-like receptor (TLR) serves as a link in innate and adaptive immunity. TLR7/8 agonist resiquimod (R848) can regulate tumor immune microenvironment by polarizing M2 macrophages into M1 macrophages and activating DC (Chen et al., 2020), further enhancing the immune system’s anti-tumor effect (Wei et al., 2021). In this study, we envisage that chemotherapy-induced ICD can be combined with other immunotherapy methods, such as inhibition of CD47 expression, a combination of R848, to regulate the immune microenvironment, enhance tumor immunogenicity, and inhibit tumor growth, recurrence, and metastasis.

Nanotechnology can improve therapeutic efficacy, increase treatment efficiency, decrease drug side effects, and efficiently deliver the right amount of ICD inducers to certain tissues or cell types (Duan et al., 2019). By improving the Enhanced permeability and retention effect (EPR effect) and altering the surface of ligands, nanoparticles can be actively or passively targeted to tumors. They can also be loaded with multiple components at once to accomplish multi-drug combination therapy, preventing the payload from being broken down and preventing the premature release of drugs. To provide individualized treatment, nanoparticles can also be tailored based on their size, shape, structure, payload, and surface characteristics (Fu et al., 2021). Briefly, nanotechnology can effectively deliver the best dose of ICD inducer to specific sites, load multiple drugs simultaneously (Fu et al., 2021), protect the payload from degradation and premature release, and realize combined immunotherapy (Duan et al., 2019; Patra et al., 2018).

Paclitaxel is the first-line drug for breast cancer, and albumin paclitaxel for injection (Albumin Bound) is the first chemotherapeutic nano drug approved by Food and Drug Administration (FDA) and widely used in the clinical treatment of breast cancer (Weaver, 2014; Abu et al., 2019). Therefore, we chose PTX as the research object to further verify that chemotherapy-induced ICD plays an important role in breast cancer immunotherapy (Wang et al., 2021). Our research group’s previous experiments have confirmed that carboxyl polyethylene glycol polylactic acid glycolic acid (PEG PLGA) nanoparticles can jointly carry and transport siCD47 and R848, and are enriched in tumor sites and draining lymph nodes, enhancing the anti-tumor effect of the autoimmune system (Li et al., 2023). Based on the above research, we combine the immunotherapy of PTX-induced ICD with siCD47 and R848 to treat the 4T1 tumor-bearing mice, verify the effect of combined immunotherapy on the growth and metastasis of breast cancer, reveal the mechanism of combined immunotherapy, and provide strong support for the effective treatment of breast cancer.

## 2 Materials and methods

### 2.1 Reagents

Dulbecco’s modified Eagle’s medium (DMEM), PBS, penicillin/streptomycin, l-glutamine, fetal bovine serum (FBS), Aqua Dead Cell Stain Kit and collagenase type IV were purchased from Thermo Fisher Scientific (Waltham, MA, United States). The RBC lysis buffer was purchased from Solarbio (Beijing, China). siRNA-targeting mouse CD47 mRNA (antisense strand, 5′-UGGUGAAAGAGGU-CAUUCCdTdT-3′) and negative control siRNA with a scrambled sequence (antisense strand, 5′-ACGUGACACGUUCGGAGAAdTdT-3′) were synthesized by Suzhou Biosyntech Co. Ltd. (Suzhou, China). The Click-iT Plus TUNEL Assay was performed for *in situ* apoptosis detection and was purchased from Thermo Fisher Scientific (MA, United States). Paclitaxel was obtained from TCI (Shanghai, China). Resiquimod (R848) was purchased from MCE (Shanghai, China). Calretinin (H-45) was peuchased from Santa Cruz (Texas, United States). ATPlite 1step assay kit was purchased from PerkinElmer (United States). Mouse HMGB1 ELISA Kit was obtained from Genin (Ireland).

### 2.2 Cell culture

The mouse breast cancer 4T1 cells, donated by the American Typical Culture Preservation Center, were cultivated in DMEM (Carlsbad, CA, United Nations) containing 10% FBS (Waltham, MA, United States) and 1% penicillin and streptomycin (complete DMEM) at 37°C in a humid atmosphere of 5% CO_2_. Bone marrow-derived DCs (BMDCs) or bone marrow derived macrophages (BMDMs) were generated by flushing bone marrow cells in the tibias and femur of C57BL/6 mice, and then culturing them in RPMI 1640 medium supplemented with 10% FBS, 10 ng/mL mouse IL-4, and GM-CSF or M-CSF for 7 days.

### 2.3 Preparation and characterization of nanoparticles

Use a micropipette to add water phase W1: 25 μL SiCD47 solution, organic phase O: 300 μL PEG-PLGA solution, 100 μL DOTAP solution, 100 μL PTX solution, and 5 μL R848 (1 mg/mL) solution into a 50 mL sterile centrifuge tube, mix well, place the centrifuge tube in an ice water mixture, sonicate at 60% power for 60 s, and stop for 1 s every 4 s to form W1/O colostrum. Add 5 mL of double distilled water (ddH_2_O) into a 50 mL centrifuge tube, mix thoroughly, place the centrifuge tube in an ice water mixture, sonicate at 60% power for 60 s, and stop for 1 s every 10 s to form W1/O/W2 emulsion. Transfer the W1/O/W2 emulsion to a round bottom flask and remove the organic solvent chloroform on a spin evaporator. Transfer the evaporated nanomedicine solution to a 50 mL centrifuge bottles polycarbonate and centrifuge at 40000 *g*, 4°C for 1.5 h. Add an appropriate amount of ddH_2_O to resuspend the centrifuged particles and makeup to 1 mL. Transfer the resuspended nanomedicine to a 1.5 mL sterile EP tube, seal, and store at 4°C.

The preparation of the control formulation is as follows:

(1) NP/Blank: For the first emulsion of water phase W1, an equal volume of ddH_2_O was used instead of siCD47 solution, and for organic phase O, 100 μL of chloroform was used instead of PTX solution and R848 (1 mg/mL) solution.
(2) NP/PTX: For the first emulsion of water phase W1, use an equal volume of ddH_2_O instead of siCD47 solution, and do not add R848 solution to organic phase O.
(3) NP/PTX/SiCD47: Organic phase O without R848 solution.

### 2.4 Loading efficiency of PTX

Take 100 μL of nanomedicine and add it to a 2 mL EP tube. Place it in a −80°C freezer to freeze it into a solid state. Turn on the freeze dryer, and after the temperature is ≤ −50°C and the pressure is ≤10Pa, sample and freeze dry. Configure the mobile phase required for high-performance liquid chromatography (HPLC) with acetonitrile: water (double distilled water) = 70:30. Filter the prepared mobile phase twice in a fume hood to remove impurities and prevent blockage of the HPLC pipeline. Ultrasonic degassing until the instrument voltage stabilizes, about 30 min. The standard curve for detecting PTX has concentration gradients of 500, 250, 125, 62.5, 31.25, and 15.625 μg/mL. Set the parameters for HPLC measurement of PTX: flow rate of 1.0 mL/min, wavelength of 227 nm, injection volume of 20 μL, and running time of 15 min for each sample. Add 1 mL of acetonitrile to a 2 mL EP tube to disintegrate the lyophilized NP/PTX, NP/PTX/siCD47, and NP/PTX/siCD47/R848 nanomedicines. Transfer 1 mL of the sample solution to an HPLC-specific solution. Detection in the sample bottle. Calculate the encapsulation efficiency (EE) of PTX in PEG-PLGA polymer nanoparticles using the following formula: EE = *W*1/*W*2 × 100% (W1 is the mass of encapsulated PTX (μg), W2 is the total mass of PTX in the system (μg)).

### 2.5 Detection of CRT expression on cell membrane

1×10^5^ 4T1 cells were cultured in 24 well plates using 1 mL DMEM complete culture medium containing 5% CO2 and a 37°C constant temperature cell incubator. Wait for the cells to adhere to the wall, discard the supernatant, add culture medium containing nanomedicine, and culture for 24–48 h. After 24 h (48 h) of drug action, discard the supernatant and wash twice with PBS. Add Calreticulin AF647 antibody diluted 1:80 and incubate at 4°C in the dark for 30 min. Wash once with FACS buffer at 1,200 rpm and centrifuge for 5 min to collect cells. Flow cytometry was used to detect and collect fluorescent signals, and Flowjo software was used to analyze the results.

### 2.6 Detection of extracellular ATP release

Collect the supernatant of the cells incubated with NPs after 24 or 48 h. Equilibrate the substrate vial and the buffer solution at room temperature before reconstitution. A water bath set at 20°C–22°C can be used for this. Reconstitute the lyophilized substrate solution by adding the appropriate volume of buffer to the substrate vial. Mix the contents of the vial by inversion and leave the solution to stand for 5 min. This should result in a clear homogeneous solution. For 96-well microplates add 100 µL of the reconstituted reagent to each well containing cells, growth factors, or cytotoxic agents to a final volume of 100 µL. Ensure that the microplate is equilibrated at room temperature (20°C–22°C) before adding the reagent. Shake the 96-well microplate for 2 min at 700 rpm using an orbital microplate shaker with an orbit diameter of 3 mm. Then measure luminescence immediately.

### 2.7 Detection of extracellular HMGB1 release

Dissolve the standard sample in a sample dilution buffer to prepare a standard solution, and dilute it by a multiple ratio. Dilute the 25 X wash solution dissolved at room temperature with ultrapure water to a 1 X wash solution. Collect the supernatant of the cells incubated with NPs after 24 h (48 h), add 100 μL of sample/standard to each well in a 96-well plate, set up three wells for each sample, and incubate at 37°C for 90 min. Wash the detergent three times, each time for 1–2 min. Add 100 μL of biotin-labeled antibody diluted 1:100 and incubate at 37°C for 60 min. Wash the detergent three times, each time for 1–2 min. Add 100 μL of SABC solution diluted 1:100 and incubate at 37°C for 30 min. Wash the detergent 6 times, 1–2 min each time. Add TMB substrate solution and incubate at 37°C in the dark for 15 min. Add 50 μL of Stop Solution and mix thoroughly. Detection of Optical Density (OD) at 450 nm wavelength using an ELISA reader.

### 2.8 Co-culture of tumor cells and BMDCs or BMDMs

Bone marrow-derived DCs (BMDCs) or bone marrow derived macrophages (BMDMs) were generated by flushing bone marrow cells in the tibias and femur of C57BL/6 mice, and then culturing them in RPMI 1640 medium supplemented with 10% FBS, 10 ng/mL mouse IL-4, and GM-CSF or M-CSF for 6 days. The 4T1 cells incubation with PBS, NP/Blank, NP/PTX, NP/PTX/siCD47, NP/siCD47/R848 for 48 h respectively were marked by 1,1′-Dioctadecyl-3,3,3′,3′-Tetramethylindodicarbocyanine, 4-Chlorobenzenesulfonate Salt (DiD) and collected for co-culture with BMDCs and BMDMs. The cells were harvested and measured by flow cytometry.

### 2.9 Animals and the tumor model

Female BALB/c mice and BALB/c nude mice (6–7 weeks old) were purchased from Charles River Laboratories (Beijing, China) and housed in a specific pathogen free environment, with free access to food and water. All animals received care following the guidelines outlined in the “Guidelines for the Care and Use of Laboratory Animals”. All programs have been approved by the Animal Care and Use Committee of Jilin University. To establish a tumor-bearing mouse model, 4T1 cells (5 × 10^5^) were suspended in 100 μL PBS and subcutaneously injected into the axilla of the mice. Determine the tumor volume (in cubic millimeters) by measuring the length (L) and width (w), and calculate it as *V = lw* ^*2*^ */2.*

### 2.10 Biological distribution of nanoparticles

NP/DiR/Cy5-siCD47, NP/DiI/Cy5-siCD47, or PBS is administered intravenously to 4T1-tumor bearing mice. The dosage of DiR (DiI) and siRNA is 1.4 mg/kg and 5 mg/kg respectively. Fluorescence image acquisition was performed using the Xenogen IVIS Lumina system (Caliper Life Sciences, USA) at 1, 2, and 24 h after injection. 24 h after injection, tissues containing tumors, lymph nodes, and other organs were collected from these mice and imaged to observe the distribution of NP/PTX/siCD47/R848. Analyze the results using Living Image 3.1 software (Caliper Life Sciences). Optimal Cutting Temperature (O.C.T.) embedding was performed on tumor and lymph node tissues. Frozen sections of the tissue were observed under confocal microscopy (Olympus FV1000, Tokyo, Japan). Zenblue 3.8 software (Zeiss) was used for analysis.

### 2.11 Therapeutic effect of NPs on 4T1-tumor bearing mice

Tumor-bearing BALB/c mice were prepared by injection of 5 × 10^5^ 4T1 cells into the mammary fat pad. When the tumor volume of mice reached about 60mm^3^, mice with similar tumor sizes were randomly divided into groups (n = 5) and were given NP/PTX/siCD47/R848, NP/PTX/siCD47, NP/PTX, NP/Blank or PBS by intravenous injection. The doses of PTX for each injection were 10 mg/kg, respectively. A total of 12 doses were administered, with the first dose on day 0 and continuous administration for 3 days. Each 3 days was considered as one course of treatment, for a total of four courses of treatment. Tumor volumes and the mouse weights were measured every day. The day after the last treatment, tissues containing the tumor, lymph node, and other organs of these mice were excised for preparation of single-cell suspension for flow cytometry assay or fixed with 4% paraformaldehyde and embedded in paraffin for H&E staining and Ki-67 or TUNEL assay. Tissue sections were visualized under a laser scanning confocal microscope (Olympus FV1000, Tokyo, Japan).

### 2.12 Statistical analysis

Perform statistics on GraphPad Prism eight and compare paired and unpaired analyses using unpaired Student’s t-test or one-way analysis of variance (ANOVA). Use the Kaplan-Meier method and time series test to statistically evaluate the survival rate of mice. *p*-value < 0.05 is considered statistically significant.

## 3 Results

### 3.1 Synthesis and characterization of nanoparticles

Polylactic acid-hydroxyacetic acid copolymers (PLGA) have been approved by FDA and are widely used for drug delivery. The coupling of poly (ethylene glycol) (PEG) with PLGA can avoid the rapid clearance of PLGA by the conditioning of body proteins and the reticuloendothelial system, and obtain good hydrophilicity to build amphiphilic copolymers PEG-PLGA NPs. The drug-carrying nano-delivery system encapsulating PTX, siCD47, and R848 simultaneously was prepared by double emulsification (Supplementary Figure S1). The well-prepared nanomedicine was used for subsequent experiments.

The surface zeta potential and hydrodynamic diameter of NP/PTX, NP/PTX/siCD47, and NP/PTX/siCD47/R848 nanopharmaceuticals were measured by dynamic light scattering (DLS). The loading efficiency of PTX in NPs was detected by HPLC.

**FIGURE 1:**
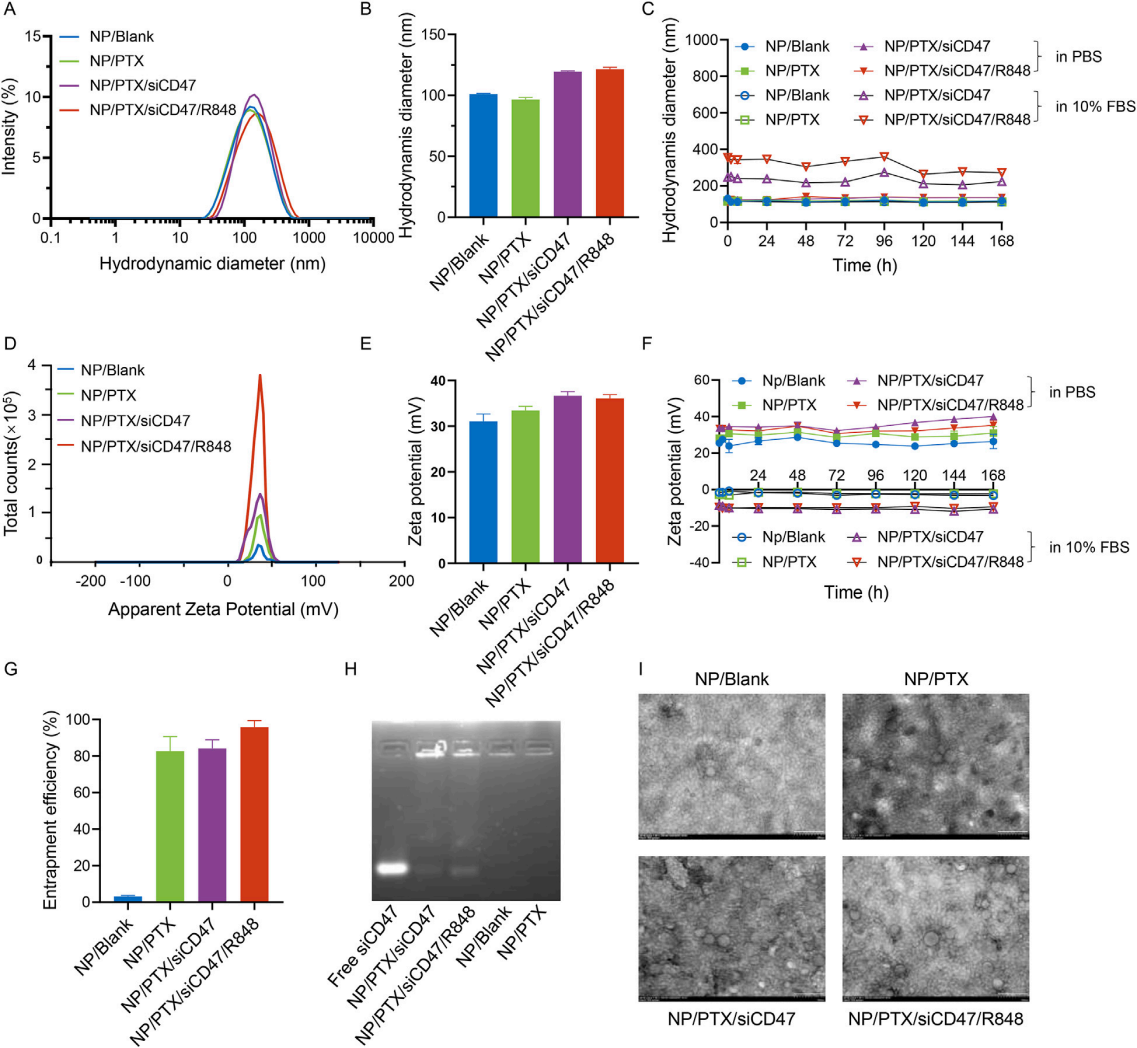
Characterization of four Nanomedicine. **(A, B)** Hydrodynamic diameters of NP/Blank, NP/PTX, NP/PTX/siCD47, NP/PTX/siCD47/R848. **(C)** Intensity distribution of drodynamic diameter of NP/Blank, NP/PTX, NP/PTX/siCD47, NP/PTX/siCD47/R848 after incubated in PBS and PBS containing 10% FBS at 37°C for 0, 2, 6 until 168 h. The diameter was measured by DLS (*n* = 3). **(D, E)** Zeta potential of NP/Blank, NP/PTX, NP/PTX/siCD47, NP/PTX/siCD47/R848. **(F)** Zeta potential of NP/Blank, NP/PTX, NP/PTX/siCD47, NP/PTX/siCD47/R848 after incubated in PBS and PBS containing 10% FBS at 37°C for 0, 2, 6 until 168 h (*n* = 3). **(G)** Loading efficiency of PTX in NP/Blank, NP/PTX, NP/PTX/siCD47, NP/PTX/siCD47/R848. **(H)** RNA agarose gel electrophoresis. The unencapsulated siRNA during the preparation of the NP/Blank, NP/PTX, NP/PTX/siCD47, NP/PTX/siCD47/R848 was measured by RNA agarose gel electrophoresis. **(I)** Transmission electron microscope (TEM) image of NP/Blank, NP/PTX, NP/PTX/siCD47, NP/PTX/siCD47/R848. Scale bar was shown in the figure. Data were presented as mean ± SEM (*n* = 5).

The hydrodynamic diameter of NP/Blank was 101.1 ± 1.5 nm on average, and the surface zeta potential was stabilized at 31.0 ± 4.9 mV. The hydrodynamic diameter of NP/PTX was 96.61 ± 5.0 nm, the zeta surface potential was 33.4 ± 2.8 mV, and the loading efficiency of PTX was 82.7%. The hydrodynamic diameter of NP/PTX/siCD47 was 119.4 ± 2.1 nm, the surface zeta potential was 36.7 ± 2.7 mV, and the loading efficiency of PTX was 84.2%. NP/PTX/siCD47/R848 had a particle size of 121.5 ± 4.5 nm, a surface potential of 36.1 ± 2.5 mV (Figures 1A, B, D, E), and a PTX loading efficiency of 95.8% (Figure 1G). The siRNA has an encapsulation efficiency of more than 95% (Figure 1H). NPs were incubated for up to 1 week in PBS or PBS containing 10% serum at 37°C, and were investigated stable under physiological conditions through DLS measurement. The slight change in hydrodynamic diameter is due to the formation of protein corona in serum, and the negatively charged NPs in serum also suggests their safety for *in vivo* application (Figures 1C, F). The morphology of the nanoparticles was all homogeneous spherical as observed by transmission electron microscope (TEM), scanning electron microscope (SEM), and atomic force microscope (AFM) (Figure 1I; Supplementary Figure S2).

### 3.2 NP/PTX induces immunogenic cell death of breast cancer cells *in vitro*

**FIGURE 2:**
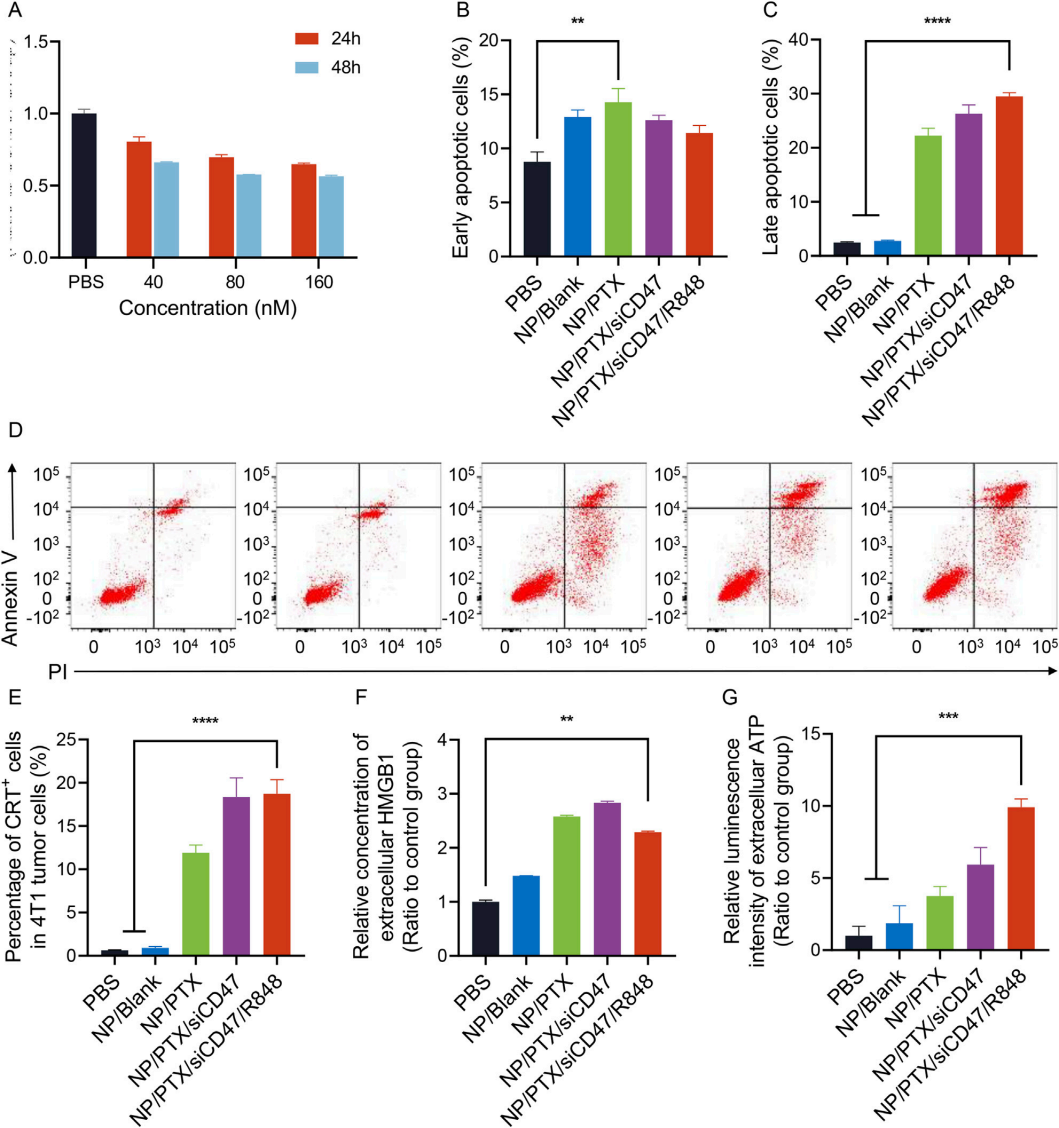
Nanoparticles loaded with paclitaxel induce ICD in 4T1 cells *in vitro*. **(A)** Nanomedicine inhibited proliferation in a time-dependent and dose-dependent manner. **(B–D)** Nanomedicine induced early and late apoptosis in 4T1 cells. Representative images of flow cytometry results are listed here. **(E)** The proportion of cells with surface CRT expression after the incubation of nanomedicine. **(F)** Relative concentration of extracellular HMGB1 after the incubation of nanomedicine. **(G)** Relative luminescence intensity of extracelluar ATP after the incubation of nanomedicine. Data were presented as mean ± SEM (*n* = 3). (*, *p* < 0.05; **, *p <* 0.01; ***, *p* < 0.001; ****, *p* < 0.0001).

Chemotherapeutic agents are known to induce immunogenic cell death of tumor cells. Our experiments show that paclitaxel-loaded nanoparticles inhibit the proliferation and induce the death of tumor cell 4T1 in a time- and concentration-dependent manner (Figures 2A–D). The half maximal inhibitory concentration (IC50) of the nanoparticles was 454.7 nM for 24h and 330.9 nM for 48 h incubation. When immunogenic cell death occurs, it is usually accompanied by changes in damage-associated molecular patterns (DAMPs) levels, which include increased cell surface CRT expression, and release of ATP and HMGB1. We observed that paclitaxel-loaded nanoparticles caused cell membrane ectopia and CRT exposure in a time- and concentration-dependent manner, accompanied by the release of ATP and HMGB1 into the supernatant (Figures 2E–G; Supplementary Figures S3A, B). Although decreased levels of ATP were detected in cell supernatants after 24 h of incubation, we suggest that this may be due to an increase in the number of cell deaths, energy depletion during the death process, and progressive degradation of ATP outside the cells (Supplementary Figure S3C)

### 3.3 NP/PTX-treated tumor cells can cause activation of BMDCs and BMDMs

Cell death is accompanied by the emission of adjuvant-like signals that promote the recruitment and activation of antigen-presenting cells. Immunogenic cell death releases DAMPs (ICD-associated DAMPs) that can be recognized by pattern-recognition receptors (PRRs), inducing APC cell activation, differentiation, and maturation. In addition, it promotes the release of type Ⅰ interferon, chemokines, and the recruitment of APCs and T cells, further activating immunity.

**FIGURE 3:**
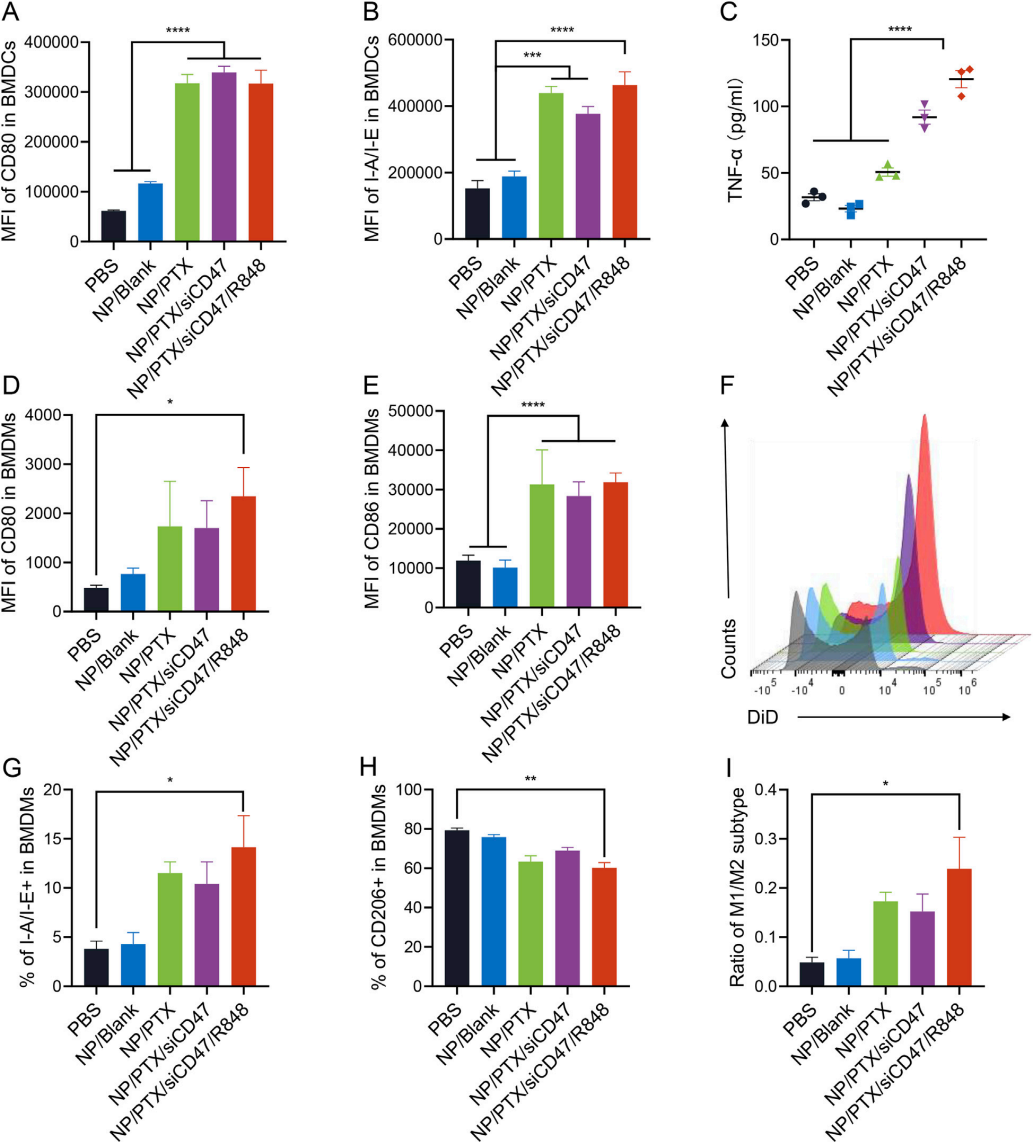
NP/PTX-treated tumor cells can cause activation of BMDCs and BMDMs. **(A, B)** Flow cytometry analysis of the surface expression of CD80 and I-A/I-E on BMDCs upon treatment with PBS, NP/Blank, NP/PTX, NP/PTX/siCD47, NP/PTX/siCD47/R848 for 48 h. **(C)** Detection of the content of TNF-α in cell supernatants of BMDCs upon treatment with PBS, NP/Blank, NP/PTX, NP/PTX/siCD47, NP/PTX/siCD47/R848 for 48 h by ELISA. **(D, E)** Flow cytometry analysis of the surface expression of CD80 and CD86 on BMDMs upon treatment with PBS, NP/Blank, NP/PTX, NP/PTX/siCD47, NP/PTX/siCD47/R848 for 48 h. **(F)** Representative histogram of the counts of intracellular DiD of BMDMs upon treatment with PBS, NP/Blank, NP/PTX, NP/PTX/siCD47, NP/PTX/siCD47/R848 for 48 h **(G–I)** The percentage of M1 or M2 subtypes and the ratio within BMDMs upon treatment with PBS, NP/Blank, NP/PTX, NP/PTX/siCD47, NP/PTX/siCD47/R848 for 48 h. Data were presented as mean ± SEM (*n* = 3). (*, *p* < 0.05; **, *p <* 0.01; ***, *p* < 0.001; ****, *p* < 0.0001).

In the present study, we found that tumor cells incubated with NPs and co-cultured with BMDCs and BMDMs that had been induced *in vitro* up to day 6 in a direct-contact manner significantly promoted the maturation and activation of BMDCs and BMDMs, resulting in elevated expression of IAIE, CD80 and CD86 and others on their surfaces. ELISA assay demonstrated increased TNF-α in cell supernatants and decreased immunosuppressive IL-6 release (Figures 3A–E; Supplementary Figure S4A).

Tumor cells typically overexpress CD47, triggering the inhibitory receptor SIRPα expressed on macrophages to evade phagocytosis and antitumor immunity. In our previous studies, it has been demonstrated that nanoparticles carrying siCD47 can downregulate CD47 expression on the surface of tumor cells (Li et al., 2023). In this experiment, we were surprised to find that after using DiD to label tumor cells treated by nanoparticles loaded with both PTX and siCD47 could act synergistically to significantly promote phagocytosis of tumor cells by BMDMs and polarize more towards M1-type macrophages with anti-tumor effects (Figures 3F–I; Supplementary Figure S4B). This potentially demonstrates that nanoparticles can be applied in an attempt to reverse the tumor immunosuppressive microenvironment.

### 3.4 Distribution of nanomedicines *in vivo*

**FIGURE 4:**
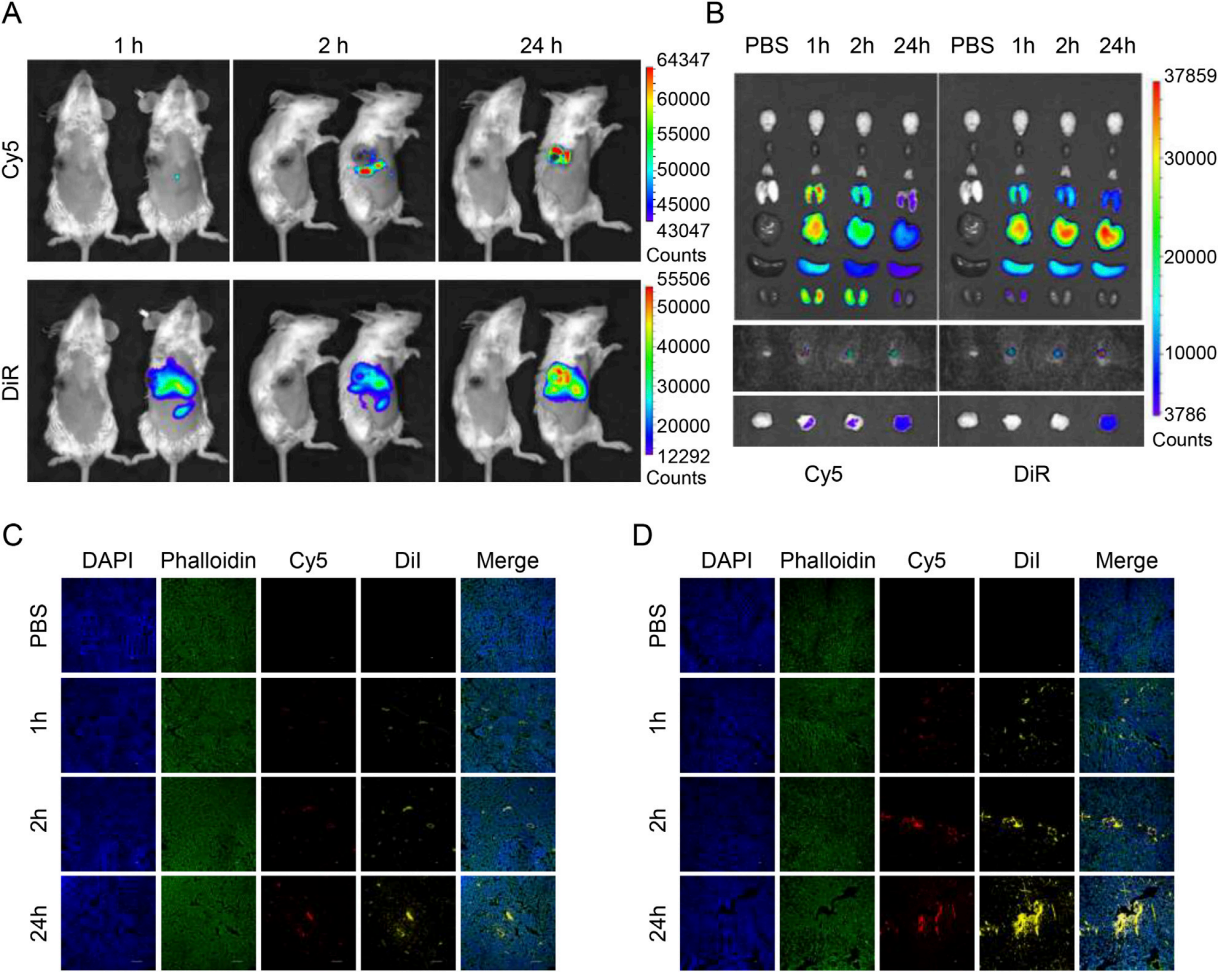
Biodistribution of drug-loaded nanoparticles in tumor-bearing mice. **(A)** *In vivo* fluorescent imaging of tumor-bearing mice after intravenous injection of NP/DiR/Cy5-siCD47 at different time points. **(B)** *Ex vivo* fluorescent imaging of the tumor and other organs. **(C, D)** Images of frozen section of tumor and draining lymph nodes taken through confocal microscope after intravenous injection of NP/DiI/Cy5-siCD47 at different time points. Data are presented as the mean ± SEM (n = 3).

We synthesized PEG-PLGA nanodrugs that can be loaded with both hydrophilic siRNAs and hydrophobic drugs, where we used Cy5 to label siRNAs while simulating lipid-soluble drugs with DiD dyes to synthesize nanoparticles that can be used for imaging *in vivo*. When the drug is injected intravenously, it takes only 2 hours to reach the tumor site, and through the EPR effect, it is continuously enriched in the tumor, and a strong fluorescence signal can still be detected until 24 h (Figure 4A).

When we collected the major organs of mice at various time points for organ imaging, we could see that the main metabolic pathways of the drugs released from the particles after circulation were slightly different, with hydrophilic drugs being metabolized more through the kidneys, while lipophilic drugs were metabolized through the liver. However, in any case, they can remain at the tumor site for a long time despite having been systemically metabolized. This implies less off-target effects, higher biosafety, and the potential to mitigate systemic toxicities of chemotherapeutic drugs (Figure 4B).

Next, we continued to use Cy5 to label the siRNA, and switched to DiI to simulate hydrophobic drugs (because of the different detectable wavelengths) to synthesize the nanoparticles, which were injected intravenously, and then the tumors and draining lymph nodes were removed and frozen sections were made. By confocal microscopy, the same trend as *in vivo* imaging could be observed, nanoparticles tended to be enriched in the tumor (Figure 4D) and draining lymph nodes (Figure 4C) with the increase of time.

### 3.5 Antitumor effects of nanomedicines *in vivo*

Murine breast cancer cell line (4T1) was *in situ* injected into mammary gland of BALB/c mice and treated by intravenous injection of NPs with PTX at a dose of 10 mg/kg per injection for a total of 12 injections (Supplementary Figure S5). The results showed that compared to the PBS control group, the NP/PTX/siCD47 and NP/PTX/siCD47/R848 groups showed a significant tumor growth inhibition on the ninth day of drug administration, the tumor size was significantly suppressed, with the volume of (562.88 ± 104.70) mm^3^ in the control group, (471.68 ± 86.80) mm^3^ in the NP/PTX group, (296.30 ± 68.22) mm^3^ in the NP/PTX/siCD47 group and (169.88 ± 61.23) mm^3^ in the NP/PTX/siCD47/R848 group.

**FIGURE 5:**
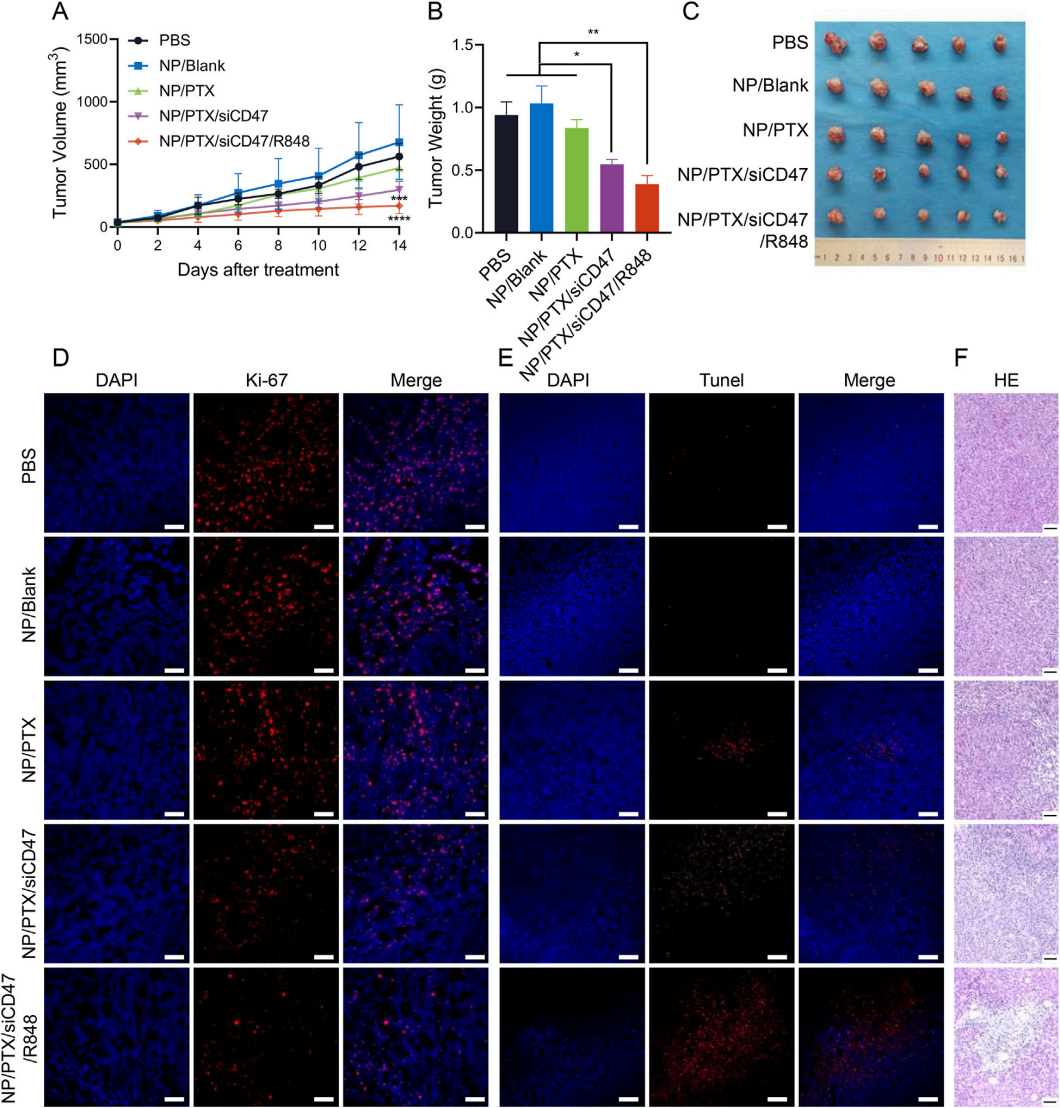
Antitumor effects of nanomedicines *in vivo*. **(A)** Tumor growth curve during each nanomedicine treatment. **(B, C)** Statistics of tumor weight and photograph of tumor extracted after completion of nanomedicine treatment. **(D, E)** Fluorescence photograph of the Ki-67 and TUNEL assay in tumor tissues after completion of nanomedicine treatment. The scale bar is 20 μm. **(F)** Microscopic image of H&E staining of main organ paraffin section. Scale bar was shown in the figure. Data are presented as the mean ± SEM (n = 5). (*, *p* < 0.05; **, *p <* 0.01; ***, *p* < 0.001; ****, *p* < 0.0001).

On day 15, all PTX-containing nanomedicines showed tumor-inhibitory effects, and the tumor-inhibitory effects of the combined treatment group were significantly higher than those of the single-drug group (Figure 5A). The mice were euthanized on day 15, and the tumors were removed and weighed. Compared with the control group, the mass of the isolated tumors was significantly reduced in each treatment group, most significantly in the combined treatment group (Figures 5B, C). The combination immunotherapy was able to inhibit the growth of mammary tumors in mice significantly.

When we performed Ki-67 and Tunel immunofluorescence staining on frozen sections of tumor tissues and observed them through confocal microscopy, we detected that all of the PTX-loaded nanomedicine treatment groups produced obvious efficacy in inhibiting tumor proliferation and inducing apoptosis, especially the combination therapy group (Figures 5D, E). Similarly, fixed and paraffin sections were subjected to H&E staining, and significant intratumor necrosis and fibrosis caused by the nanomedicine could be clearly observed by optical microscopy (Figure 5F). Furthermore, flow cytometry analysis of tumor tissue revealed an increase in CRT expression on the surface of tumor cells after treatment (Supplementary Figure S6A). These visually demonstrated the excellent anti-tumor efficacy of nanomedicines *in vivo*.

### 3.6 Nanomedicines improve the immune environment *in vivo*

**FIGURE 6:**
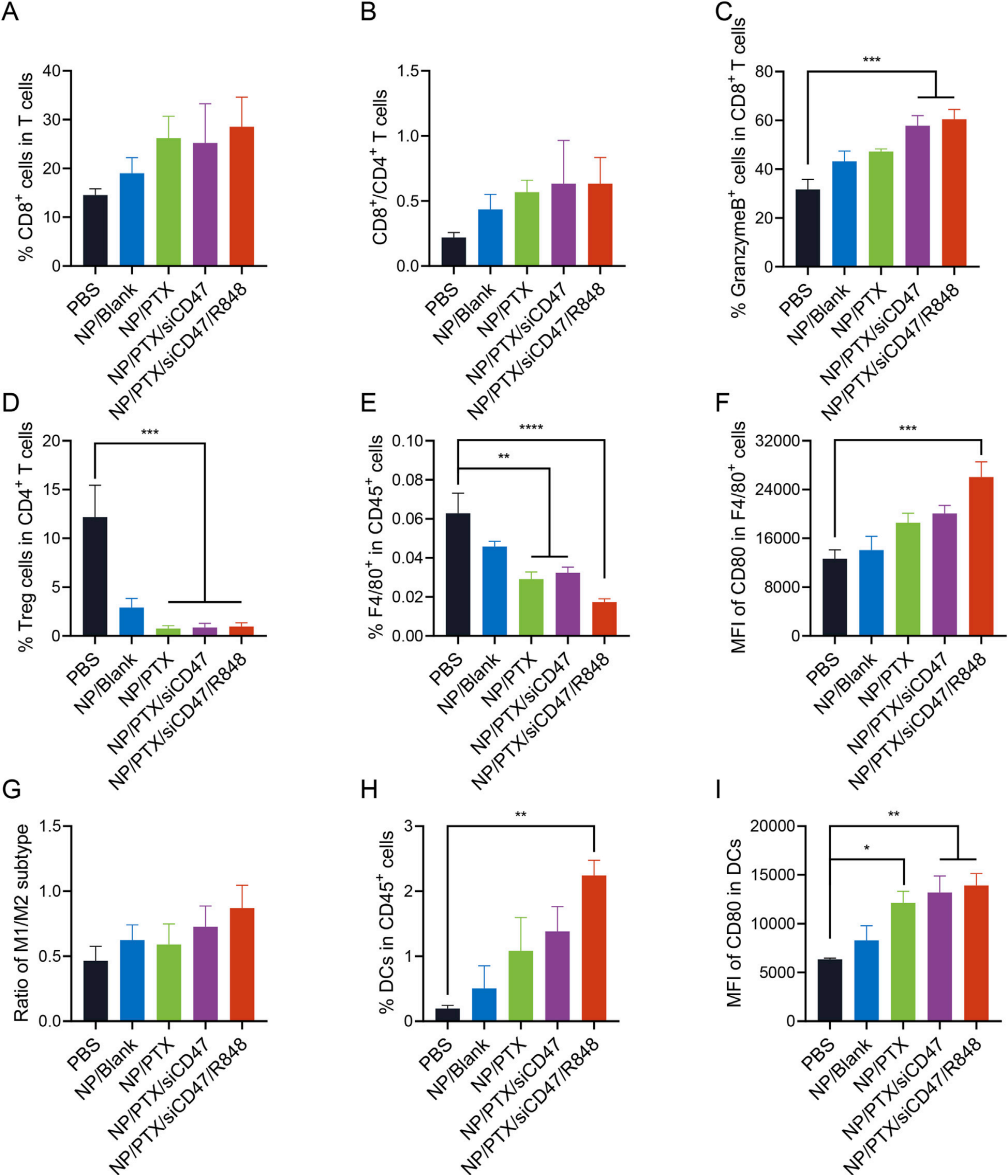
Antitumor immune response of nanomedicines *in vivo*. **(A, B)** Percentage of cytotoxic T lymphocytes (CD8^+^ T cells) and their ratio to T helper lymphocytes (CD4^+^ T cells). **(C)** Percentage of granzyme B^+^ cells in cytotoxic T lymphocytes. **(D)** Percentage of regulatory T cells (Tregs) in T helper lymphocytes. **(E, F)** Percentage of Tumor-associated macrophages (TAMs) (CD11b^+^ F4/80^+^ cells) and flow cytometry analyzed relative MFI of the surface expression of CD80. **(G)** Ratio of M1 and M2 subtype of TAMs. **(H, I)** Percentage of DCs (CD11b^+^ CD11c^+^ cells) and flow cytometry analyzed relative MFI of the surface expression of CD80. Data are presented as the mean ± SEM (n = 5). (*, *p* < 0.05; **, *p <* 0.01; ***, *p* < 0.001; ****, *p* < 0.0001).

In order to explore whether the nanomedicine NP/PTX/siCD47/R848 can inhibit tumor growth by modulating the immune response, the tumors, tumor-draining lymph nodes (TDLN), spleens, and peripheral blood of treated mice were taken, and the percentage of different kinds of immune cells was analyzed by flow cytometry. The results showed that in tumor tissues, the CD8/CD4 ratio was significantly higher in the NP/PTX/siCD47 and NP/PTX/siCD47/R848 treatment groups compared with the control group and the PTX single-agent nanoparticle group, and the increase in the secretion of granzyme B by the CD8^+^ T cells also reflected their highly efficient activation level, suggesting a potent activation of the adaptive immune system (Figures 6A–C; Supplementary Figure S6B), whereas the elevation of the percentage of B cells in the spleen also flanks the intrinsic immune system was activated (Supplementary Figure S6C). Even more strikingly, treatment with nanomedicines resulted in a substantial depletion of Treg (Figure 6D). At the same time, there was a trend towards a decrease in tumor-associated macrophages and MDSC (Figure 6G and Supplementary Figure S6D), which comprise the immunosuppressive microenvironment, and the surviving macrophages were significantly activated and had the potential to polarize into the M1 subtype (Figures 6E–G; Supplementary Figures S6E, F). In the peripheral blood, the percentage of DC cells and the expression of CD80 co-stimulatory factor on DC cells were significantly higher in the NP/PTX/siCD47 and NP/PTX/siCD47/R848 treatment groups compared with the control group and the NP/Blank group, reflecting the massive recruitment of APCs in the periphery after nanoparticle treatment (Figures 6H, I).

**FIGURE 7:**
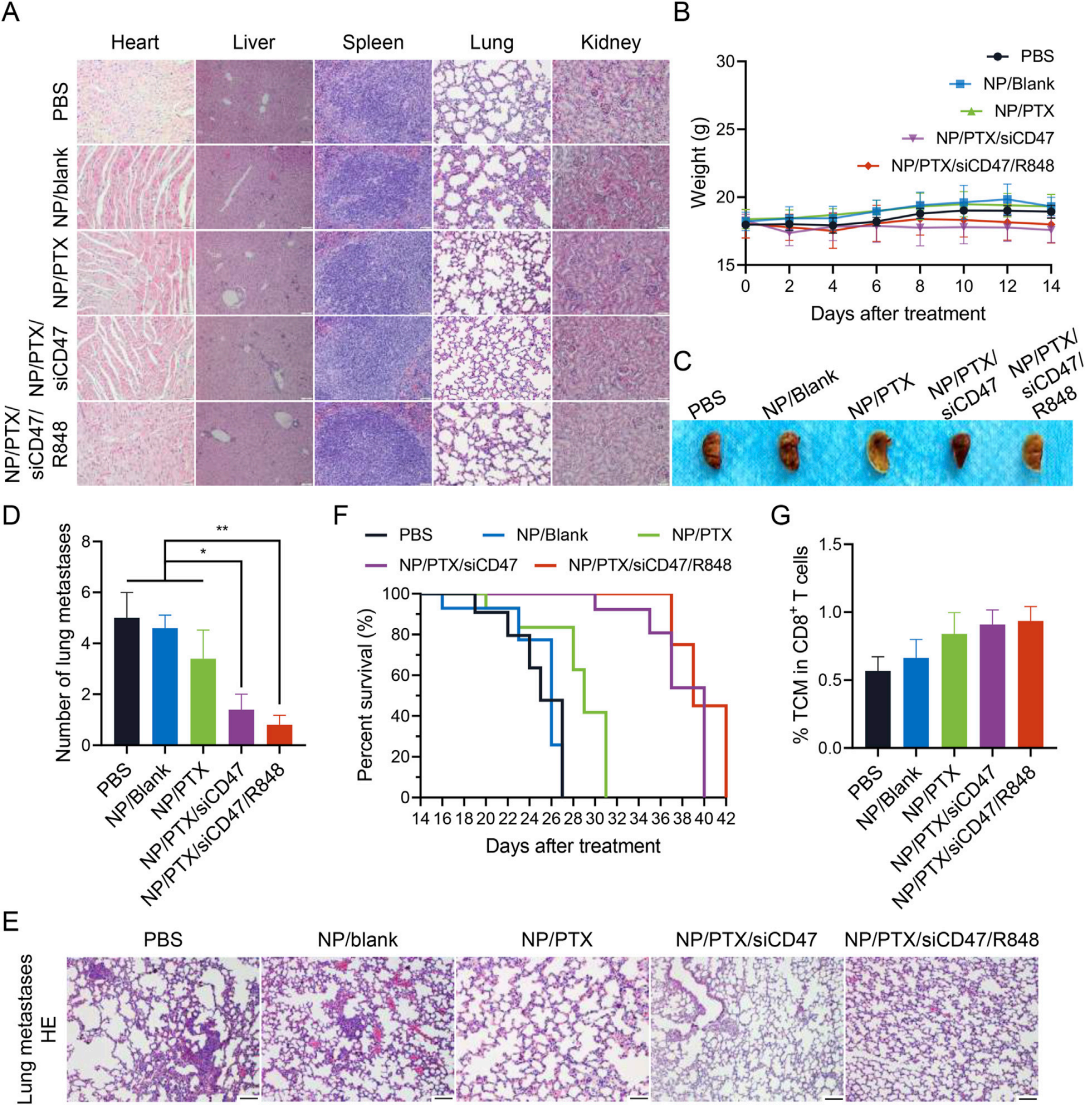
The safety and anti-tumor metastasis ability of nanomedicine therapy *in vivo*. **(A)** Microscopic image of H&E staining of main organ paraffin section. Scale bar was shown in figure. **(B)** The body weights of 4T1 tumor bearing mice during each nanomedicine treatment. **(C)** Macrophotographic images of lung metastasis after nanomedicine treatment. **(D)** Microscopic image of H&E staining of lung metastasis after nanomedicine treatment. **(E)** Statistics of the number of lung metastatic nodules after nanomedicine treatment. **(F)** Survival period of mice in each group after nanomedicine treatment. **(G)** Percentage of central memory T cell (TCM) in cytotoxic T lymphocytes. Data are presented as the mean ± SEM (n = 5). (*, *p* < 0.05; **, *p <* 0.01).

Under the premise of ensuring anti-tumor efficacy, the safety of the drug is also crucial. Throughout the treatment cycle, no obvious wasting was observed in the mice (except for the natural consumption caused by the development of tumors) and when the important organs were removed for paraffin sectioning and H&E staining, it could be seen that the organs were still able to maintain intact morphology without any obvious damage (Figures 7A, B). Observation of the lung lobes and the statistical analysis of the number of pulmonary metastatic nodules seems to explain the possible reasons for the risk of mortality and poor prognosis in mice in later life (Figures 7C–E; Supplementary Figure S7). Next, we observed the survival of the mice after treatment with the same administration method and cycle, and the graph shows intuitively that although the nanomedicine treatment cannot completely cure the tumors, it can still effectively improve the PFS. It is worth noting that this improvement in survival is more due to the introduction of siCD47 and R848 than to the chemotherapeutic effect of PTX alone (Figure 7F). Flow cytometric analysis shows the increased percentage of memory T cells predicted its possible anti-recurrence and anti-metastasis potential (Figure 7G). This demonstrates that the synergistic effect of immune therapy and chemotherapy-induced ICD can effectively improve the tumor microenvironment and enhance the response-ability of the systemic immune system. This not only inhibits tumor growth *in situ* but also has the potential to reduce distant metastasis and improve overall prognosis.

## 4 Discussion

Paclitaxel is indispensable for neoadjuvant and adjuvant chemotherapy of breast cancer during clinical treatment (Geyer et al., 2022). However, the toxic side effects caused by chemotherapy seriously affect the quality of life of patients (Azim et al., 2011). In theory, the toxic side effects of chemotherapy can be alleviated by reducing drug dosage, targeting drug delivery to the tumor site, and other methods (Gradishar et al., 2005). The low immunogenicity of breast tumors is one of the reasons for high-dose chemotherapy and actively exploring chemotherapy-induced tumor immunogenic cell death is expected to reduce the effective dose of chemotherapy drugs (Wang et al., 2024). At present, the research on paclitaxel-induced ICD in breast cancer is relatively scarce and mostly stays at the cellular level (Zhai et al., 2023). Therefore, this study focuses on the impact of PTX on tumor immunity in the treatment of breast cancer.

This study confirmed that PTX can increase the expression of CRT on the surface of the 4T1 cell membrane of breast cancer cells and induce the ICD effect of breast cancer, which is consistent with the previous research results of Wang Ying and others (Wang et al., 2021; Duan et al., 2021). However, due to the low immunogenicity of breast tumors and the presence of an immunosuppressive microenvironment, it is difficult to reverse the immunosuppressive microenvironment with a single medication. Therefore, we choose to combine other immunotherapies. Chen et al.'s study showed that PTX liposomes combined with anti CD47 (aCD47) effectively synergistically inhibit the proliferation and metastasis of TNBC tumor cells (Chen et al., 2021). R848 can improve the tumor immunosuppressive microenvironment. Based on this, we chose siCD47 and R848, which have previously successfully achieved combined delivery, to be used in combination with PTX.

This study successfully prepared a nano drug delivery system containing PTX, siCD47, and R848 using a double emulsification method. Prior to this, research on nano drug delivery systems was mostly limited to the combination of two drugs. Our animal experimental results indicate that the nanomedicine NP/PTX/siCD47/R848 can effectively inhibit the growth of breast tumors *in situ*. The inhibition of breast tumor growth by NP/PTX/siCD47/R848 may be achieved by increasing the proportion of CD8^+^ T cells in the tumor microenvironment and promoting their activation; Simultaneously reducing the proportion of Treg and MDSC weakens the immunosuppressive microenvironment. Furthermore, it is possible to recruit APCs from the periphery and induce their differentiation and maturation, enhance antigen extraction ability, stimulate the formation of memory T cells, and regulate anti-tumor therapy through innate immune conditions, in order to reduce distant metastasis and improve long-term prognosis. Undoubtedly, DC cells play an important role in anti-tumor immunity (Gardner and Ruffell, 2016). This study found that NP/PTX/siCD47/R848 enhances anti-tumor immunity by promoting DC expression. However, the role of CD4^+^ T in tumor immunity remains to be explored. A deeper understanding of the specific mechanisms and functional regulation of these cytotoxic CD4^+^T cells is expected to provide effective immunotherapy methods for populations that do not respond to current immunotherapies (Oh and Fong, 2021).

It is certain that chemoimmunotherapy using chemotherapeutic agents that directly kill tumor cells to induce ICD to achieve enhanced anti-tumor immunity is feasible, and CD4^+^ T-cell-based immunotherapy is also an effective strategy for controlling tumor progression and recurrence, and our experiments have expanded the options for tumor immunotherapy. However, the advancement of these immunotherapeutic approaches has a long way to go. On the one hand, despite the proliferation of new drugs that induce ICD, the complete mechanism, transport in the organism, and metabolism of various ICD inducers need to be further investigated. On the other hand, a deeper understanding of the biology of CD8^+^ T cells is needed. In addition, models and biomarkers capable of predicting the expected efficacy of combinations in clinical trials need to be developed to enable easy and accurate assessment of the therapeutic potential of different combinations of drug modalities at the same level, predicting therapeutic response (Plana et al., 2022), clinical response to treatment-resistant therapies, patient survival (Tesi, 2019), and to achieve a match between tumor biology, the immune system, and therapeutic mechanisms.

In summary, this study demonstrated that paclitaxel can induce immunogenic death of breast tumors, and the nanomedicine NP/PTX/siCD47/R848 can significantly inhibit the growth of breast tumors *in situ*. The mechanism of these effects is closely related to the regulation of the ratio of multiple immune cells in the immune microenvironment of tumors to improve tumor immunogenicity. Although the specific molecular mechanism has not yet been revealed, it is not clear whether the same efficacy can be achieved in clinical trials. However, based on the current study, we believe that chemotherapy-induced ICD combination therapy will shortly be gradually transformed into clinical practice, which will provide strong support for individualized immunotherapy for breast cancer and make precision therapy possible.

## Data Availability

The original contributions presented in the study are included in the article/Supplementary Material, further inquiries can be directed to the corresponding authors.
