# Supplementary material for: Nanomedicine based on chemotherapy-induced immunogenic death combined with immunotherapy to enhance antitumor immunity
Source: Front Pharmacol. 2024 Dec 4;15:1511423. doi: 10.3389/fphar.2024.1511423 (PMC11652165; doi:10.3389/fphar.2024.1511423)
Supplement: Supplementary file 1 [file DataSheet1.docx]

Supplementary Material

# Supplementary Figures

#
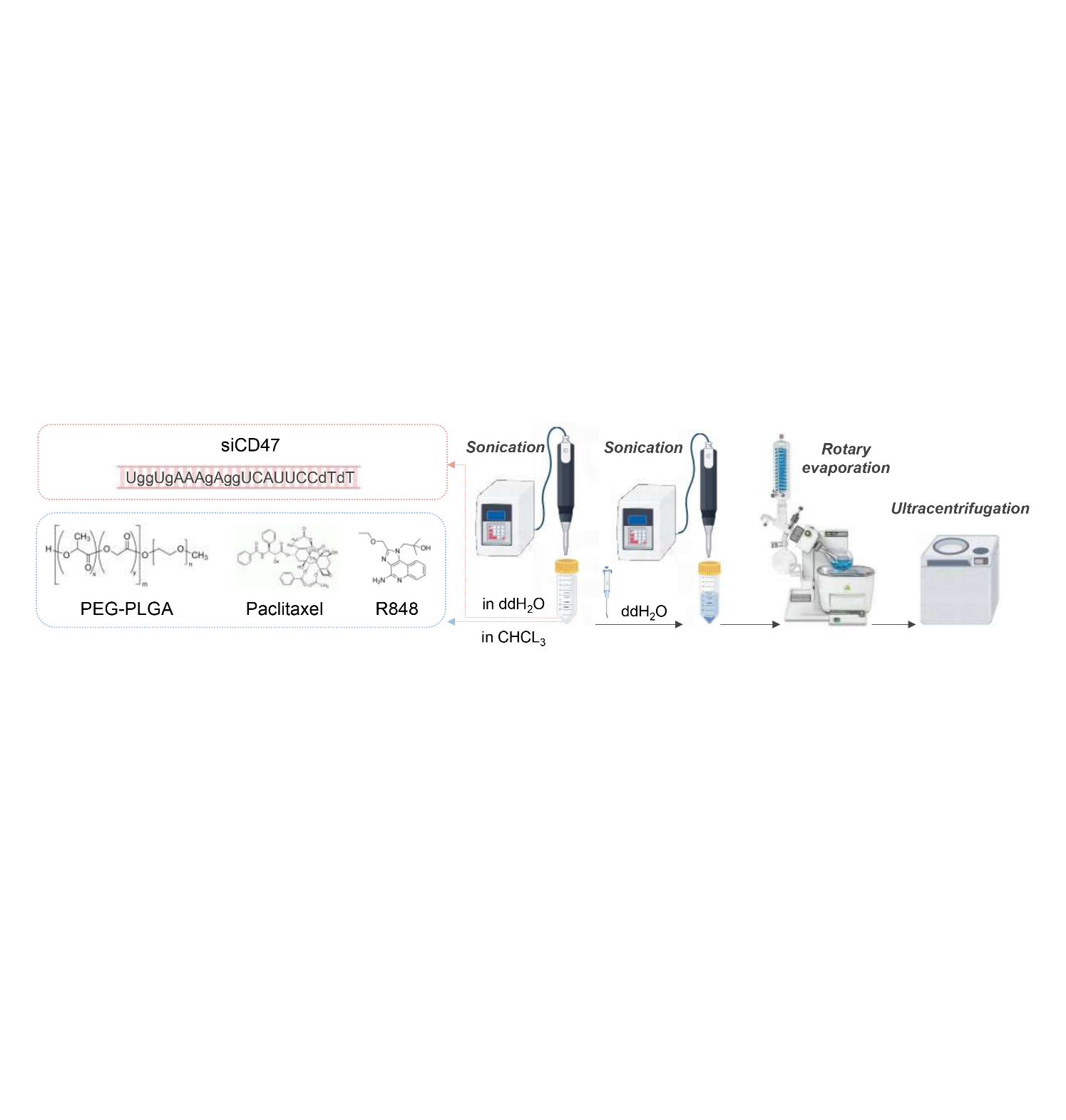


**Supplementary Figure 1.** Preparation process of nanomedicine. Sonicate and mix the water phase W1 (SiCD47 solution) and organic phase O (PEG-PLGA solution, DOTAP, PTX solution, and R848 solution) for first emulsification. Sonicate the ddH_2_O and the mixture above for second emulsification. After rotary evaporation of the mixed solution, ultracentrifugation is performed to obtain nanomedicine.

**
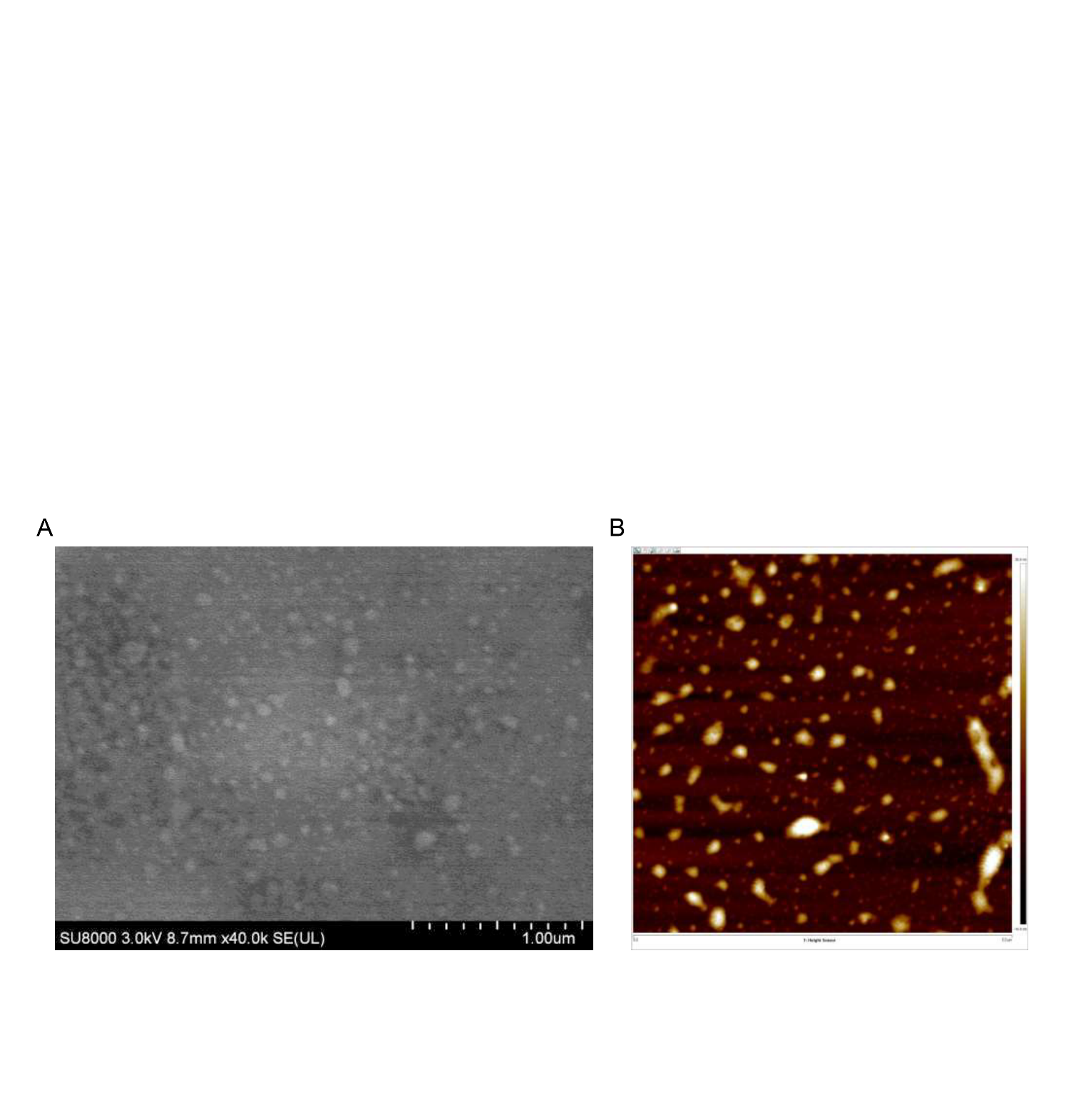
**

**Supplementary Figure 2.** (A) Scanning electron microscope (SEM) image of NP/PTX/siCD47/R848. Scale bar was shown in the figure. (B) Atomic force microscope (AFM) image of NP/PTX/siCD47/R848. Scale bar was shown in the figure.


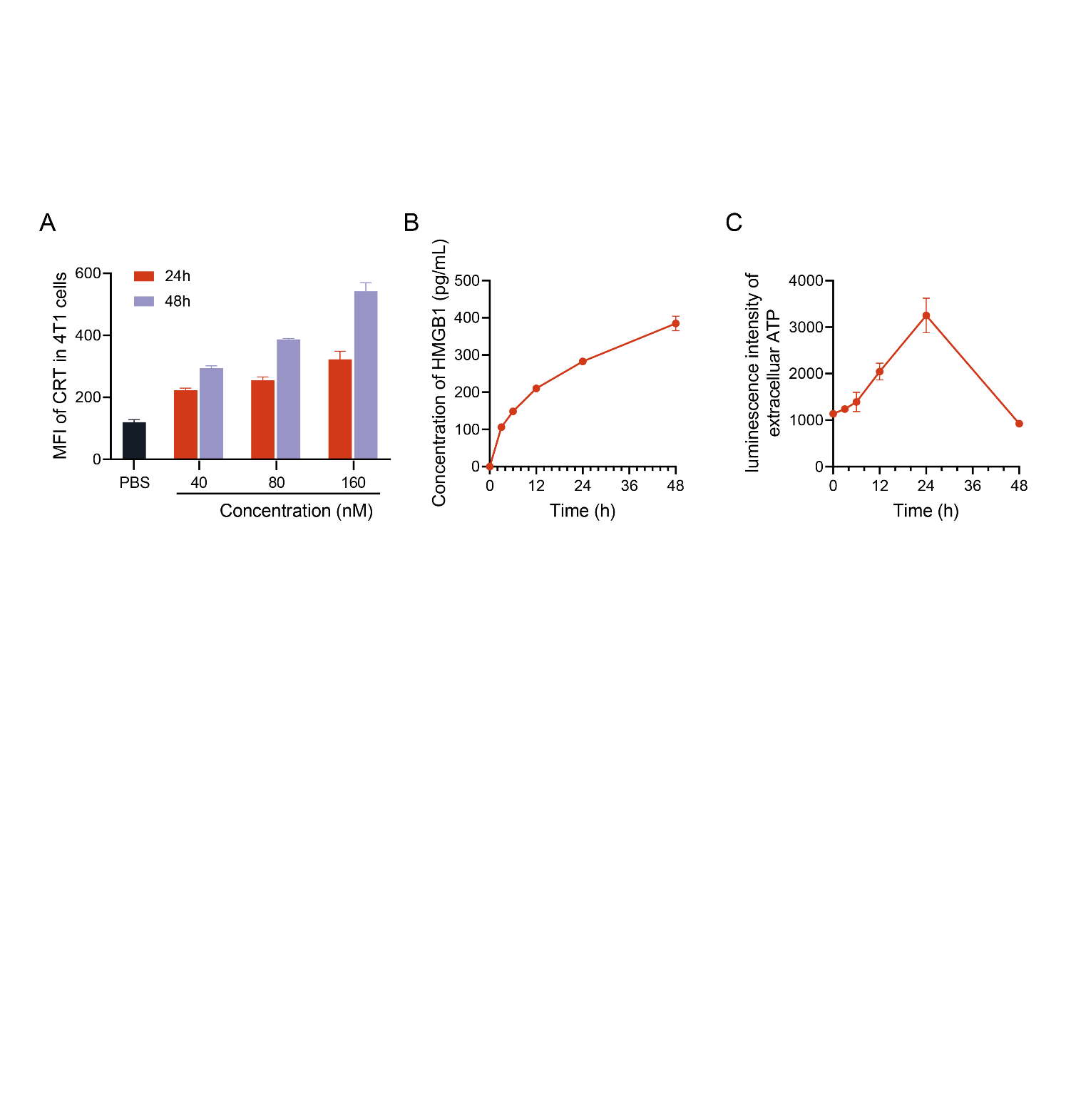


**Supplementary Figure 3.** (A) Nanomedicine increased MFI of the surface CRT of 4T1 cells in a time-dependent and dose-dependent manner. (B) Concentration of extracellular HMGB1 after the incubation of nanomedicine for different time. (C) Luminescence intensity of extracellular ATP after the incubation of nanomedicine for different time. Data are presented as the mean ± SEM (n = 3).


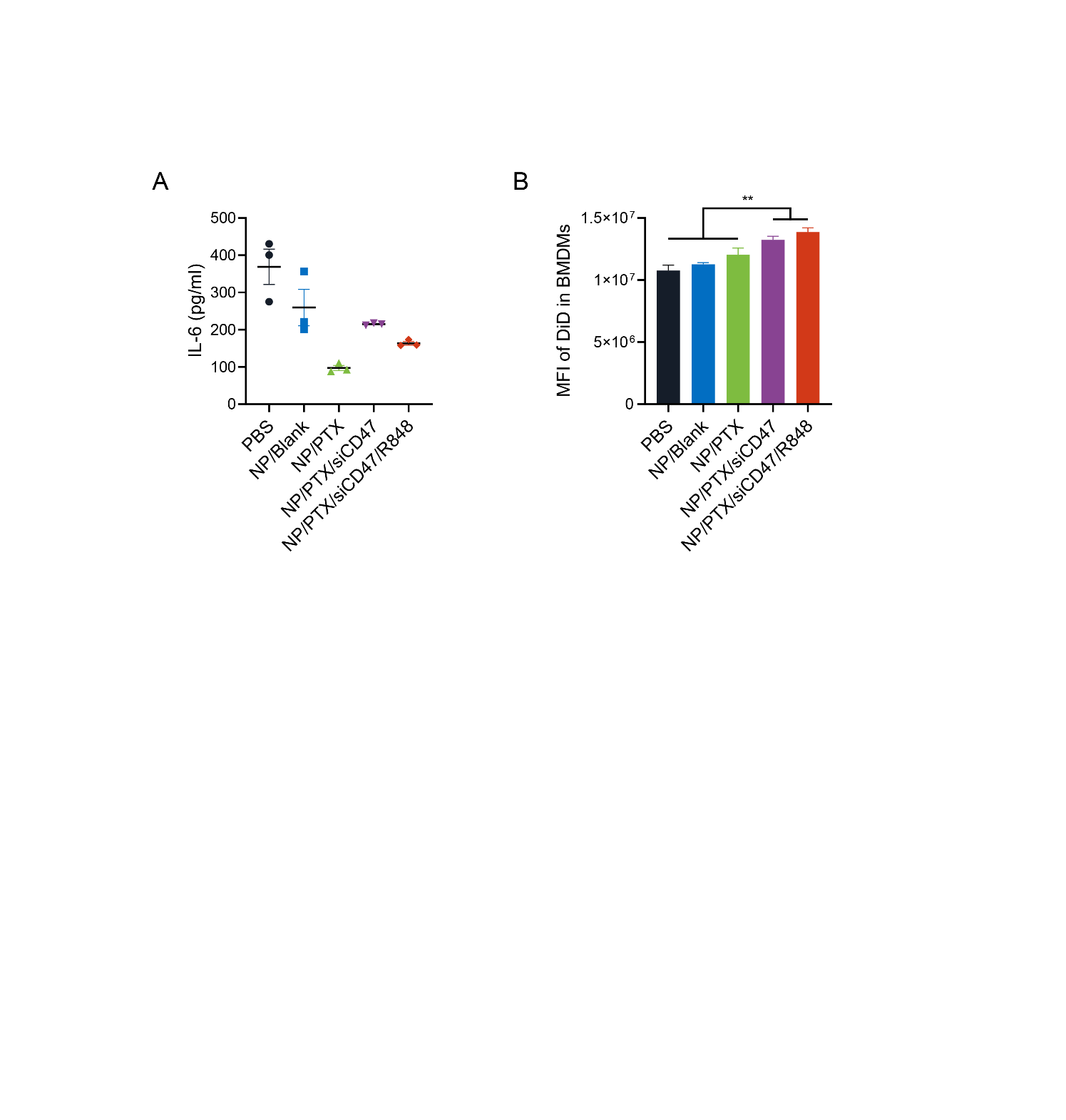


**Supplementary Figure 4.** (A) Detection of the content of IL-6 in cell supernatants of BMDCs upon treatment with PBS, NP/Blank, NP/PTX, NP/PTX/siCD47, NP/PTX/siCD47/R848 for 48 h by ELISA. (B) Flow cytometry analysis of the MFI of DiD in BMDMs upon treatment with PBS, NP/Blank, NP/PTX, NP/PTX/siCD47, NP/PTX/siCD47/R848 for 48 h. Data are presented as the mean ± SEM (n = 3). (**, *p <* 0.01)


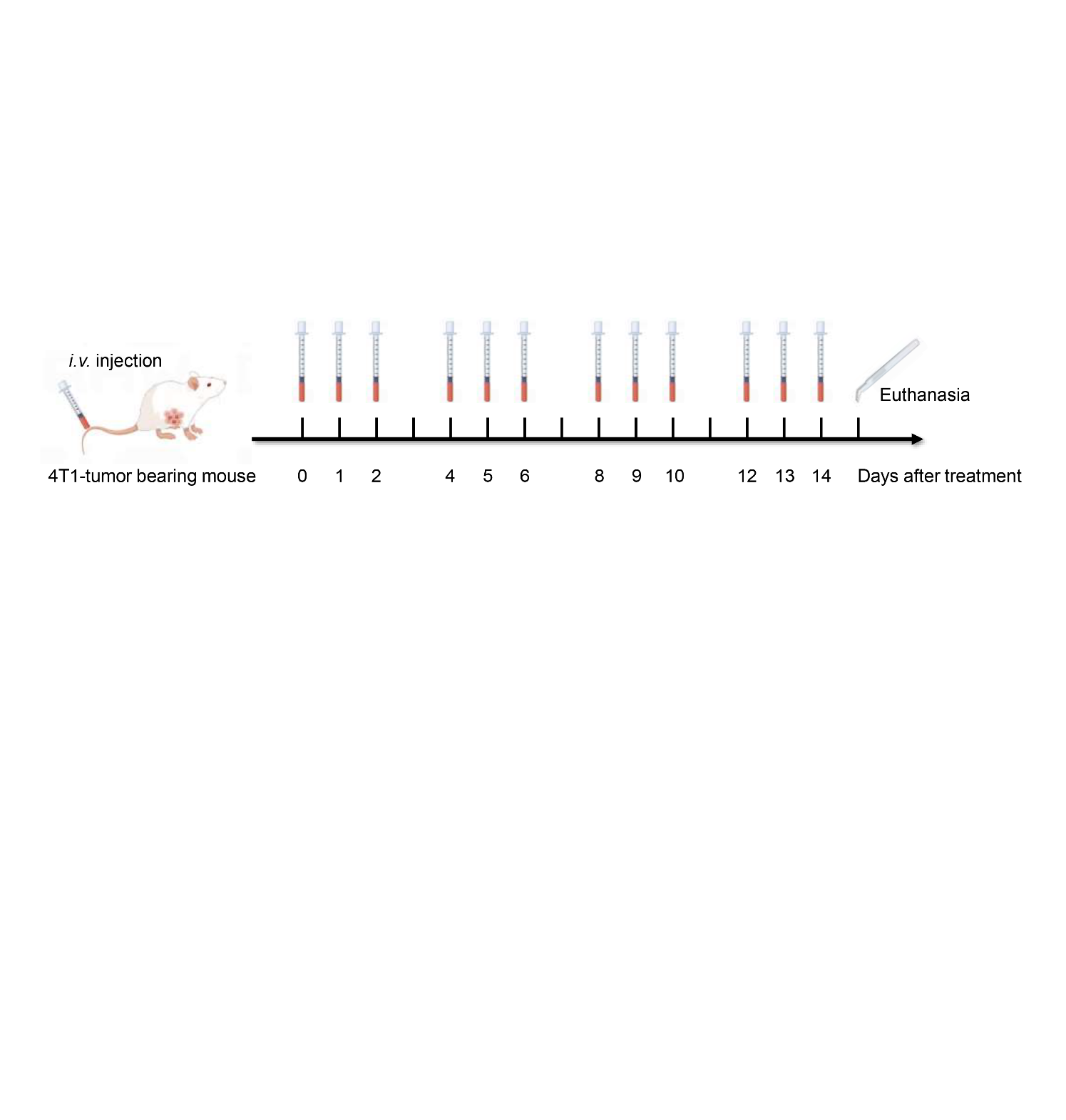


**Supplementary Figure 5.** Therapeutic schedule of nanomedicine for 4T1 tumor-bearing mice. A total of 12 doses were administered, with the first dose on day 0 and continuous administration for 3 days. Each 3 days was considered as one course of treatment, for a total of 4 courses of treatment. Tumor volumes and the mouse weights were measured every day. The day after the last treatment, tissues containing the tumor, lymph node, and other organs of these mice were excised for the following experiments.


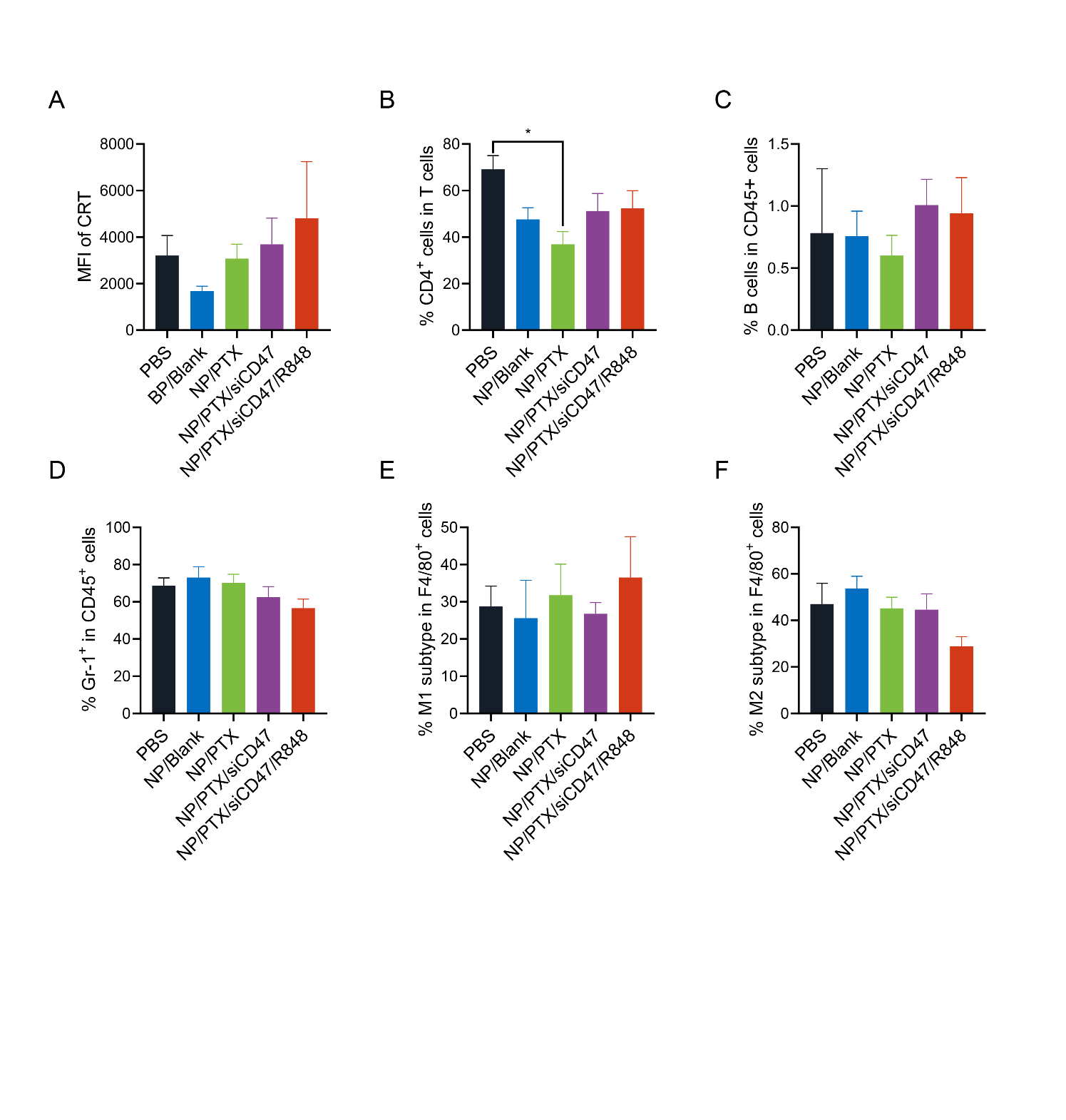


**Supplementary Figure 6.** Anti-tumor immune response of nanomedicines *in vivo*. (A) Flow cytometry analyzed relative MFI of the surface CRT of tumor cells after nanomedicine treatment. (B) Percentage of T helper lymphocytes (CD4^+^ T cells) in T lymphocytes. (C)Percentage of B cells (CD45^+^ CD19^+^ cells) in immune cells within the tumor microenvironment. (D) Percentage of myeloid-derived suppressor cells (MDSCs) (CD11b^+^Gr-1^+^) in immune cells within the tumor microenvironment. (E F) Percentage of M1 or M2 subtype of TAMs within the tumor microenvironment. Data are presented as the mean ± SEM (n = 5). (*, *p* < 0.05)


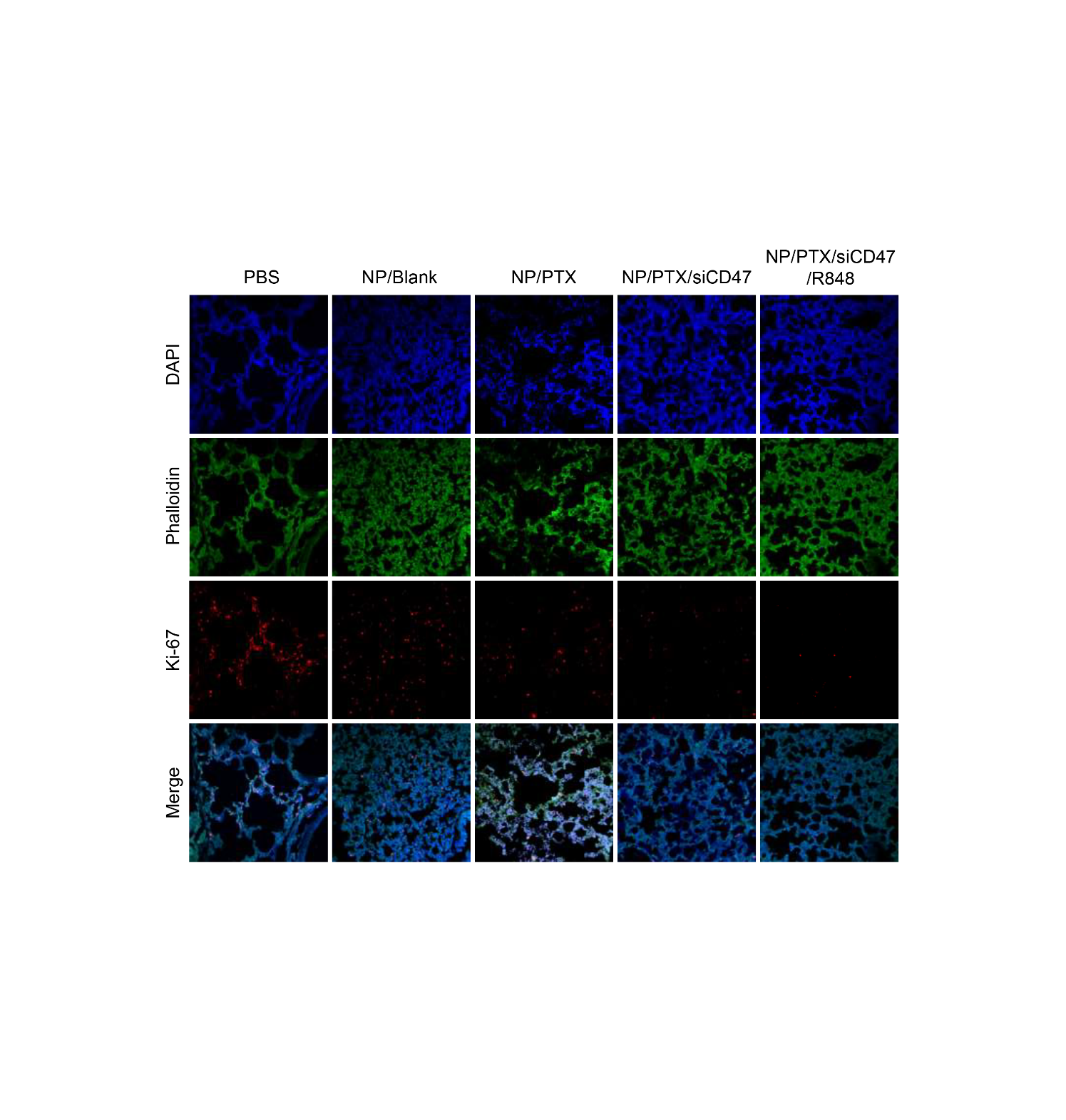


**Supplementary Figure 7.** Fluorescence photograph of the lung metastasis after nanomedicine treatment, Ki-67 staining is used for tumor identification. The scale bar is 20 μm.
